# Turning the tide on big cat trade: Expert opinion on trends and conservation lessons from the Republic of Korea

**DOI:** 10.1371/journal.pone.0299783

**Published:** 2024-05-15

**Authors:** Joshua Elves-Powell, Hang Lee, Jan C. Axmacher, Sarah M. Durant

**Affiliations:** 1 Institute of Zoology, Zoological Society of London, London, United Kingdom; 2 Department of Geography, University College London, London, United Kingdom; 3 Tiger and Leopard Conservation Fund in Korea, Seoul, Republic of Korea; 4 College of Veterinary Medicine, Seoul National University, Seoul, Republic of Korea; 5 Faculty of Environmental and Forest Sciences, Agricultural University of Iceland, Keldnaholt, Iceland; Cheetah Conservation Fund, Namibia University of Science and Technology, NAMIBIA

## Abstract

Unsustainable trade in big cats affects all species in the genus, *Panthera*, and is one of the foremost threats to their conservation. To provide further insight into the impact of policy interventions intended to address this issue, we examine the case study of the Republic of Korea (South Korea), which in the early 1990s was one of the world’s largest importers of tiger (*Panthera tigris*) bone and a major manufacturer of tiger-derived medicinal products. In 1993, South Korea became a Party to the Convention on International Trade in Endangered Species (CITES) and introduced a ban on commercial trade in CITES Appendix I-listed big cats a year later. We used an expert-based questionnaire survey and an exploration of the CITES trade database to investigate what has since happened to big cat trade in South Korea. Expert opinion suggested that big cat trade has likely substantially reduced since the early 1990s, as a result of the trade ban and broad socioeconomic changes. However, illegal trade has not been eradicated entirely and we were able to confirm that products reportedly derived from big cats were still publicly available for sale on a range of Korean online marketplaces, sometimes openly. The items most commonly reported by respondents from post-1994 trade and supported by expert-led evidence were tiger and leopard (*Panthera pardus*) skins and tiger bone wine. Although South Korea may provide a useful case study of a historically significant consumer country for tiger which has made strong progress in addressing unsustainable levels of big cat trade within a short period of time, there remains a need to address recalcitrant small-scale, illegal trade. We also recommend further investigation regarding reports of South Korean nationals being involved in illegal trade in tiger-derived products in Southeast Asia.

## Introduction

The global trade in big cats, whether resulting from the exchange of their bodies, body parts and derivatives, or live individuals, is geographically widespread and encompasses a diverse range of species and uses, across many different countries and cultures [1–7]. The tiger (*Panthera tigris*) is considered to be particularly threatened by unsustainable trade on account of its high desirability, especially for use in many traditional Asian medicines (TAMs) [8–11], but trade is now recognised to be an important concern for all species in the genus, *Panthera* [12], and a wide range of other felids are also known to be impacted [13]. The Felidae are subject to a family-wide Convention on International Trade in Endangered Species (CITES) Appendix II listing, with a number of species, including most big cats, listed under an Appendix I classification, which prohibits all commercial trade [14].

On the Korean Peninsula, the domestic use and trade of tiger and leopard (*Panthera pardus*), which are both native species, has a long history. There is good historical evidence for trade during the Joseon dynasty (A.D. 1392–1897), predominantly domestic tribute to the royal court, or international tributary trade and diplomatic gifts, of skins [15]. These were highly valued and following the Later Jin (1627) and Qing (1636–1637) invasions, the number of leopard skins required as tributary trade to China was increased from 50 skins, to 142 in 1637 [16]. Foreign envoys were also personally gifted with tiger and leopard skins [17] and there is some evidence that they were, at least occasionally, provided with tiger meat, bones and other body parts [18]. There is also evidence for domestic use of bones for traditional Korean medicine (TKM), although prevalence of this practice is not clear [see, for example, 19]. Despite government attempts to limit private trade in big cat parts during the Joseon dynasty [15], administrative reports show that regulations were often circumvented [20]. In the late 19^th^ century, tiger and leopard parts were used by Korean nobles (yangban) as ornaments (teeth and claws) and blankets for carrying chairs (skins), as well as in Korean and Chinese traditional medicinal tonics and pills (bones) [21–25]. Following the opening of the country’s ports to international trade in 1876, Korea also exported large quantities of tiger and leopard parts [26]. In 1887 alone, 210 tiger and leopard skins were exported from the port of Incheon, as well as a small quantity of tiger bones [27]. Tiger skins were observed by early Western travellers to initially be plentiful and cheap, but within a few decades, to have become scarce and substantially more expensive [25].

Japanese annexation of Korea in the early 20^th^ century resulted in the specific targeting of tigers by large carnivore control programmes and trophy hunters, leading to the extirpation of the Amur tiger (*P*. *t*. *tigris*) from the southern half of the Korean Peninsula by 1924 [15]. Although also targeted, the Amur leopard (*P*. *p*. *orientalis*) persisted through independence and the Korean War (1950–53), but was extirpated in 1970 [28]. The Republic of Korea (henceforth, South Korea) was not an early signatory to CITES and up to 1993, legally imported hundreds of kilogrammes of tiger bones every year [29], as well as large quantities of derivatives [30], making it one of the world’s largest markets for tiger bone in the late 20^th^ century. Customs records from 1970 to 1993 show that South Korea imported at least 8981 kg of tiger bone during this period [8] and although this was received from 12 different countries, almost half came from Indonesia [31]. At this time, South Korea was also one of two major global producers, along with China, of manufactured tiger-derived products for use in TAMs [32].

In 1993, South Korea became a Party to CITES and introduced a ban on commercial trade in CITES Appendix I-listed big cat species and their body parts a year later, largely motivated by concerns over the threat of potential trade restrictions to the United States of America [31]. This was achieved by amending several different pieces of existing legislation, of particular note being revisions to the Law Concerning the Protection of Wildlife and Game (1986, revised 1994), subsequently replaced by the Wildlife Protection and Management Act (2005, revised 2011), and the Pharmaceutical Affairs Act (1953, revised 1994), the latter dealing with international trade in endangered species for medicinal purposes and which, since 1994, included specific prohibition of the import, storage, trade or display of tiger bone and derived products. This took effect in 1994 (production) and 1995 (sale, storage and exhibition of products). Despite the size of the South Korean market for tiger in the early 1990s, there has been little research since into South Korean trade in either tiger specifically, or felids generally. Kang and Phipps [31] found that just 5.5% of 256 TKM practitioners they interviewed admitted to using tiger bone after 1994, although this figure may underrepresent the true extent of the use of tiger products at the time, given that it was based on voluntary self-reporting of potentially illegal activity [31]. While international seizure records for tiger were reported from other former and current tiger range countries, as well as from a wide range of countries where tigers have never been present, there were no recorded seizures of tiger in South Korea between 2000 and 2018 [33].

This presents two main contrasting possibilities. The ban could have succeeded in its aims and trade in body parts and derivatives of tiger and other big cats collapsed. If this was the case, then South Korea could provide a useful example of a country that was successful in implementing an effective trade ban in a short time period. Alternatively, trade could have gone underground and undocumented. These scenarios are not mutually exclusive, so it is also possible that trade widely collapsed, but with a low level of undetected trade continuing. To address this information gap, we undertook an expert elicitation survey in 2022–23, combined with an exploration of the CITES trade database. We focus on two key research questions. First, what is the expert consensus on changes in South Korean big cat trade since CITES accession and introduction of a trade ban? Second, is there expert-led evidence for ongoing illegal trade?

## Materials and methods

We conducted an expert-based questionnaire survey between October 2022 and March 2023 to document informed opinion and associated evidence of trade in big cats, their body parts and derivatives in South Korea since 1994. Expert elicitation using a structured questionnaire survey or interviews is a commonly utilised research technique for gathering information on illegal wildlife trade [34–37], because it can be used to collect information from grey literature that may not appear in formal reports or publications, and can be used to ascertain expert opinion on the reasons behind observed trends. Given the lack of experts on big cat trade in South Korea specifically, experts consulted came from broader relevant areas of expertise, namely big cat ecology and conservation, wildlife trade policy (including CITES implementation), customs administration (specifically animal import or export), law enforcement, and captive animal regulation. The inclusion of a small number of ‘external’ (international) experts on big cat trade specifically, wildlife trade generally, or big cat conservation, was intended to help identify any international trade links and to reduce the potential for groupthink by providing additional information and perspectives independent of ‘internal’ experts, who may be more likely to have shared information sources [38].

In order to identify potentially suitable participants, the authors first contacted individuals who we knew to be knowledgeable on the target subject areas, as well as major conservation organisations and government agencies with expertise in those areas and whose work potentially covered South Korea (for example, organisations which had a South Korean office or regional programmes that covered the East Asia region). Individuals contacted, whether they chose to participate in the study or not, were asked for recommendations of suitable participants.

The 38 individuals contacted were provided with a copy of the questionnaire, in Korean or English, and were given time to consider whether they had any relevant information on the topics covered. Of these, 14 respondents provided informed written consent to take part in the study, which met the typical sample size requirements for expert elicitation [37,39]. 16 respondents declined to participate, stating that they did not have sufficient information on big cat trade in South Korea to do so; 8 individuals did not respond or were unable to do so within the time constraints of this study. Although the available pool of experts was relatively small, the targeted approach to recruitment achieved a higher response and participation rate than studies which have drawn from a larger expert pool [35]. The questionnaire survey was self-completed by participants and consisted of a series of linked questions, eliciting knowledge and opinions of respondents pertaining to the occurrence of big cat trade and any trends observed since the introduction of the 1994 trade ban.

It is important to acknowledge that our findings, where based on the results of an expert elicitation, may reflect the perceptions of respondents, rather than necessarily being an accurate representation of actual trends or levels of trade [34]. Particular care should be taken given the relatively small expert pool that was available to draw from, encompassing varying backgrounds and levels of expertise [40]. Nevertheless, this approach remains a valuable way to gather preliminary information on illegal trade, as a complex and dynamic system, where information is scarce and often reliant on sporadic, informal reports [34,35,37]. In order to substantiate perceptions of trade reported by respondents, we asked for any supporting evidence that they were able to provide. This was used to contextualise and, where appropriate, challenge the responses received. We identify those responses that lack evidential support.

We combined this with an analysis of records from the CITES Trade Database (https://trade.cites.org/) of legal trade in big cats to South Korea over the past 30 years. Although there are well-documented issues with the CITES trade database, such as the failure of some countries to regularly submit reports to CITES, the compilation of records across multiple years as a single report, and frequent data gaps [34,41,42], the database remains the best publicly available information source on legal, international trade in listed species [34]. South Korea is not subject to any CITES recommendations to suspend trade on account of non-compliance [43], nor has it been at any point over the past 15 years, which may give added confidence that records for South Korea are an accurate reflection of legal trade.

We searched the CITES trade database for records of imports to, and exports from, South Korea of big cats of the genus *Panthera*. Records were checked for any inconsistencies and tabulated. We excluded trade in live animals from our primary analysis, in order to distinguish this form of trade from our primary focus, trade in big cat bodies, body parts and derived products. However, as this information may be valuable for future investigations, we present aggregated data for live trade. There were occasional discrepancies in import and export records of live animals. In such cases, we used the value reported by the importer, as export records may indicate the number of permits issued, rather than the number of live animals which were actually exported [44]. We also excluded CITES trade records for scientific specimens from our analysis, as, in the case of big cats, these are typically non-destructive. CITES data were analysed over temporal scales, in order to reveal changing patterns of legal trade in big cat bodies, body parts and derivatives to South Korea since 1994. Given the difficulty of accurately interpreting CITES records which use the source code ‘I’ (Confiscated or seized specimens), as this source code officially refers to legal trade of products that have previously been seized or confiscated [44], but is applied by CITES Management Authorities in various circumstances [45], we obtained additional information on such records from the relevant CITES Management Authority.

Ethical approval for the study was received from the ZSL Human Ethics Committee (Approval Number ZSLHEC01).

## Results

### CITES records of big cat trade

**Fig 1:**
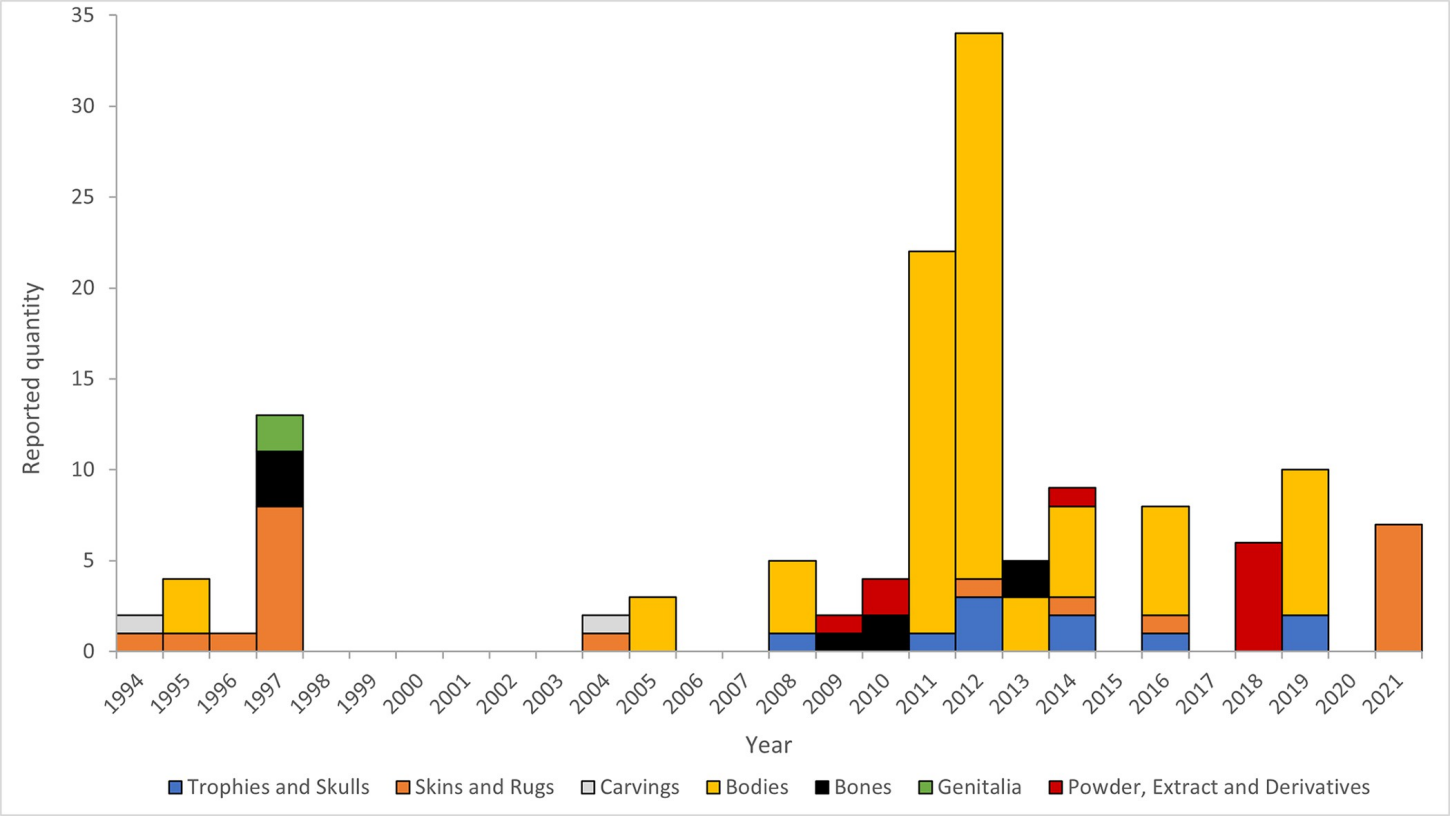
CITES registered imports of big cat (genus: *Panthera*) body parts and derived products to South Korea, 1994–2021. Compiled from the CITES Trade Database. Note, does not include live animals or scientific specimens.

CITES data on legal imports of *Panthera* bodies, body parts and derivatives to South Korea since 1994 show small quantities of inbound trade immediately after CITES accession (1994–7), which then dropped off entirely, with no legal trade from 1998–2003 and 2006–7 (Fig 1). Imports of *Panthera* species increased again from 2008, with a wide range of products being sporadically imported after this year, including trophies, skins, bodies, bones, powder, derivatives, extract, skulls and rugs. These were predominantly tiger, leopard or lion (*Panthera leo*) in origin, but also including small quantities of jaguar (*Panthera onca*). However, bodies represent by far the largest, as well as the most regularly recorded, single component of resurgent inbound trade between 2008–21 (Fig 1). There were particularly sizable imports of big cat bodies in 2011 and 2012, comprised of lion (33 animals), tiger (11), leopard (5) and jaguar (3).

CITES data on exports from South Korea for the same period, 1994–2021, were dominated by scientific specimens and live animals, which we excluded from our analysis as it focussed on trade in *Panthera* bodies, body parts and derivatives. Of the 45 further CITES records of 9769 big cat-derived products that were exported from South Korea from 1994 onwards, 22 entries refer to trade in 8422 products which were officially listed as having previously been seized or confiscated (source code ‘I’), exported to the United States of America (18 individual entries in the CITES trade database, occurring between 1994–2016) and New Zealand (4 entries, occurring between 2004–2009). Several of these exports to the United States were extremely large in quantity: in 1996, there was an entry for 7040 tiger derivatives originating from South Korea, while in 2014, 1200 tiger-derived medicines were received. Discussion of these records with former and current officials of the CITES Management Authorities for the United States and New Zealand confirmed that these two large shipments to the United States were, in fact, records of illegal imports, while all four entries from New Zealand were confirmed to have been illegal imports without valid CITES documentation.

As well as the United States and New Zealand, exports from South Korea to Japan, China, Mongolia and South Africa were also recorded. In trade records involving these countries, the source, if known, was usually given as specimens taken from the wild (W) or pre-convention specimens (O), along with one case of an animal bred in captivity (C) and one animal bred in captivity that did not fulfil the definition of ‘bred in captivity’ (F).

CITES-registered trade in live big cats to South Korea is also recorded by the CITES trade database and we compiled this separately (Table 1). The number of live animals imported (244) and exported (223) to South Korea between 1994 and 2021 was very similar, with big cats mainly being imported for zoos (135), circuses or travelling exhibitions (75), or unspecified commercial purposes (25).

**Table 1:** Legal imports and exports of live big cats (genus: *Panthera*) to South Korea,1994–2021.

	Imports	Exports
**Total**	**244**	**223**
**Zoos (Z)**	135	139
**Circuses or travelling exhibitions (Q)** **Commercial (T)**	75 25	42 20
**Scientific purposes (S)**	2	5
**Breeding in captivity or artificial propagation (B)**	1	16
**Unrecorded**	6 (USA)	1 (USA)

Compiled from the CITES Trade Database.

### Respondent demographics and extent of knowledge

16 respondents (5 from South Korea, 11 international) from 11 different organisations, including relevant South Korean government agencies and some of the world’s leading non-governmental organisations with expertise in wildlife trade, declined to participate in the survey, stating that they had insufficient information relevant to big cat trade in South Korea.

**Fig 2:**
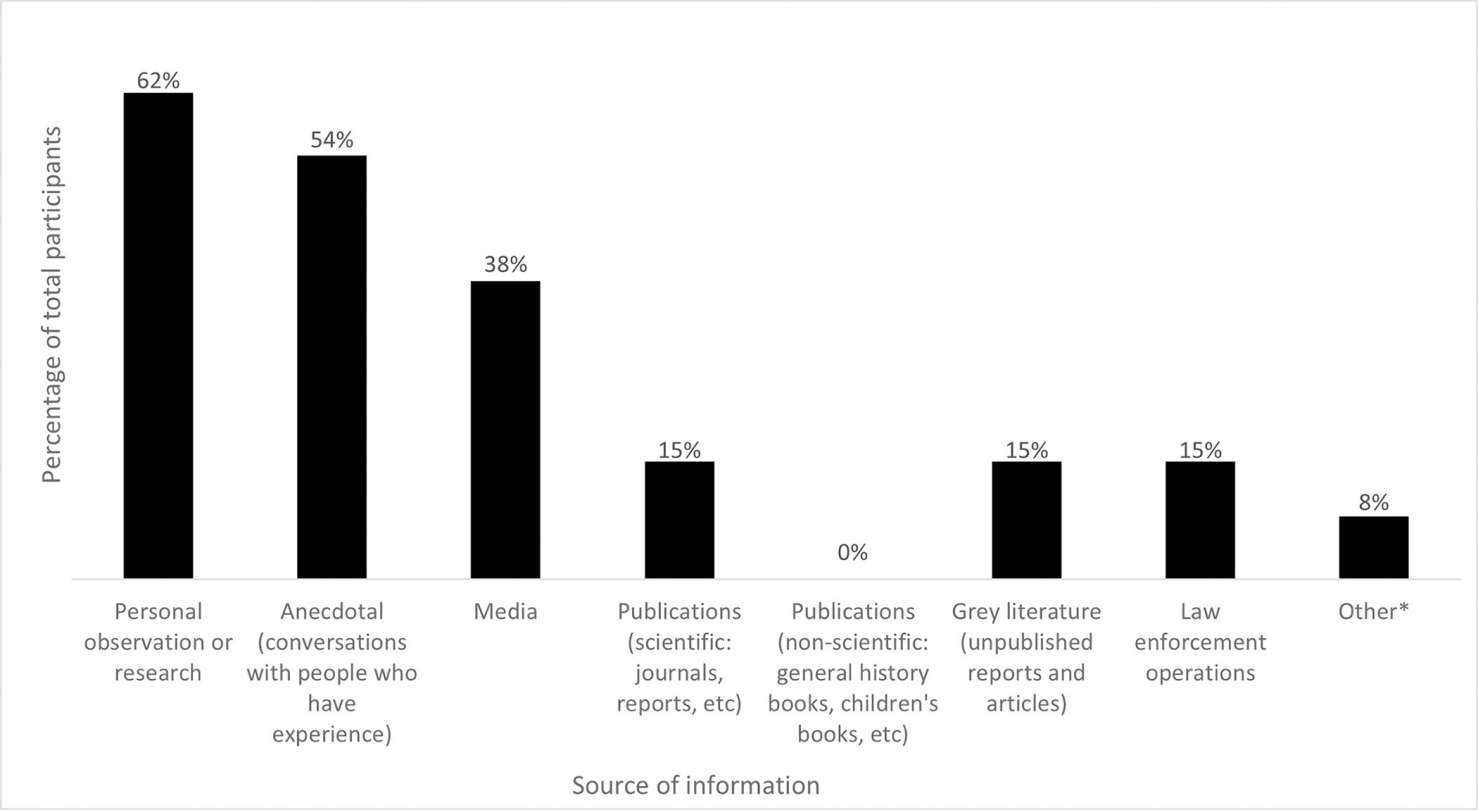
Respondents’ sources of information for big cat trade in South Korea. Includes multiple responses. *Other included CITES Trade Database.

The majority of the 14 survey participants worked in South Korea (79%), with the most common professional backgrounds being roles in government, science and research, or law and law enforcement (Table 2). The main information sources were personal observations or research, as well as anecdotal information (for example, conversations with people who had experience of big cat trade in South Korea) and to a lesser extent, media reports, scientific publications, unpublished ‘grey’ literature and law enforcement operations (Fig 2).

**Table 2:** Professional background of expert respondents.

	Expert participants
**Total**	**14**
**Government**	5
**Non-governmental organisations**	1
**Science and Academia**	3
**Applied conservation**	1
**Law and Law enforcement**	3
**Zoo and captive animal facilities**	1

**Fig 3:**
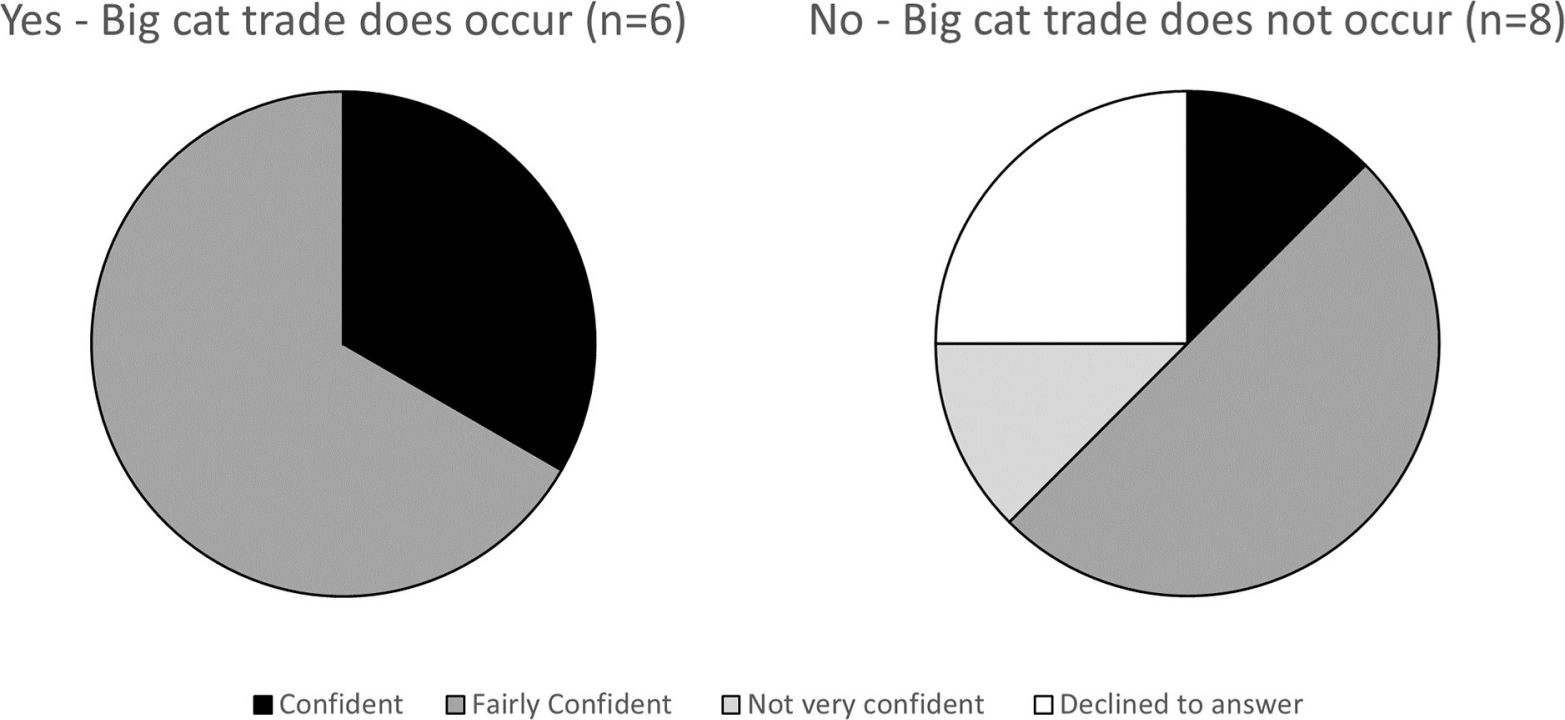
Respondents’ confidence in their response regarding occurrence of big cat trade in South Korea.

Expert participants were asked to evaluate confidence in their knowledge regarding the occurrence of big cat trade; almost all respondents reported that they were ‘confident’ or ‘fairly confident’ (Fig 3). Although responses to subsequent questions, which asked for more specific details about trade, generally indicated a greater degree of uncertainty, several respondents who had emphasised that they did not feel particularly confident in their knowledge about big cat trade in South Korea proceeded to present strong evidence to support their answers.

### Awareness of trade

Less than half of all respondents (43%) reported that trade in big cat bodies, body parts or derivatives currently occurs in South Korea. No respondents directly reported that they believe ‘large-scale’ illegal trade in big cats, including trade involving organised criminal groups, currently occurs in South Korea. Several respondents specifically mentioned extirpation of South Korea’s last big cat population in 1970 and the country’s geographical isolation, with its only land border being one of the world’s most heavily militarized, as long-term barriers to illicit trade. Respondents suggested a range of reasons for a perceived decline in big cat trade since the 1990s, which we discuss below.

Among those who reported current trade in big cats, some participants provided information from the early 1990s, when big cat trade was still legal and therefore less relevant to current patterns of trade. However, other respondents provided strong evidence that some trade still occurs. Tiger and leopard were the species most commonly mentioned, although one respondent mentioned African lion body parts from South Africa, another mentioned individual examples of clouded leopard (*Neofelis sp*.) and snow leopard (*Panthera uncia*) skins being smuggled into South Korea, and another respondent mentioned trade in jaguar and ocelot (*Leopardus pardalis*), the latter a medium-sized felid native to the Americas.

Respondents who reported that trade in big cat body parts currently occurs in South Korea mentioned a range of big cat-derived products and uses. Traditional medicine (including TKM and traditional Chinese medicine, TCM) (n = 5 responses; 35.7% of respondents) was the most commonly cited reason for big cat trade. Specific items that were mentioned by respondents are presented in Table 3, with the most commonly reported tiger-derived items being skins (n = 4), bone and its derivatives (such as tiger bone glue and tiger bone gel) (n = 4), and bone wine (n = 3), which we recorded separately from other bone-derived products. Other products which were mentioned by multiple respondents were tiger teeth (n = 2) and leopard skins (n = 3). As well as use in traditional medicine, these corresponded to use of big cat products as jewellery and personal decoration, home decoration, antiques (of unknown age or origin), and traditional uses relating to superstitions (for example, as good luck charms). Following leads provided by expert respondents, we were able to confirm that products reportedly derived from big cats were still publicly available for sale on a range of Korean online marketplaces, sometimes openly. These items were exclusively advertised as individual items (for example, a single bottle of tiger bone wine) and were typically found on online marketplaces and personal blogs.

**Table 3:** Felid body parts that expert participants reported being traded in South Korea.

Species	Product mentioned in trade	Number of times mentioned by expert participants	Evidence of post-1994 trade supplied	Further information
*Panthera tigris*	Tiger bone and derivates (tiger bone gel, tiger bone glue), excluding tiger bone wine.	4		Origin: various, including Thailand. Buyers from mainland China and Hong Kong.
	Tiger bone wine	3	Yes	Origin: China, North Korea (early 2000s), possibly from Southeast Asia.
	Skins	4	Yes	Origin: various, including Thailand. One enforcement case (2010).
	Teeth	2	Yes	Arrest in Vladivostok, Russia of a Vietnamese national following attempted transportation to Incheon, South Korea (2020) (final destination unknown).
	Claw pendant	1	Yes	
	Whiskers	1		
	Body parts (various)	1		
*Panthera pardus*	Skins	3	Yes	Seizure following transit through Incheon, from Kenya to China (2013). Confiscation by Seoul Customs of two leopard skins from Gabon (1996).
	Body parts (various)	1		
*Panthera leo*	Carcasses	1		Origin: South Africa.
*Panthera onca*	Body parts (various)	1		
*Panthera uncia*	Skin	1		Origin: Nepal (2005). Displayed at workplace.
*Neofelis* sp.	Skin	1		Origin: Thailand (2001). Displayed at home.
*Leopardus pardalis*	Body parts (various)	1		

Evidence of post-1994 trade refers to participant submissions only.

The source of products, where given, was always another country, consistent with the fact that South Korea no longer has any extant big cat populations. Countries specifically mentioned were China, Thailand, Vietnam, Russia, Nepal, Kenya and Gabon. For example, one respondent gave the example of an individual they observed trying to sell a leopard skin, which the individual claimed they had obtained whilst working in West Africa, on a well-known Korean online marketplace. South Korea was reported to be both a consumer and transit country for international trade (with end users in mainland China and Hong Kong). Expert-led evidence confirmed that big cat products destined for China have illegally transited through South Korea.

Several respondents discussed products being misidentified as felid in origin. For example, one respondent provided an example where an individual asked for identification of a souvenir that they had purchased in China and which they believed to be tiger in origin. However, DNA analysis revealed it to be derived from banteng (*Bos javanicus*) [46]. Another respondent discussed a ‘leopard’ skin that was confiscated by Seoul Customs, but which was subsequently identified as a dog skin dyed to look like a leopard.

### Decline in trade since the 1990s

A major theme was the belief that big cat trade had declined substantially in South Korea since the early 1990s, reflected by a reported lack of awareness by the majority of respondents of any trade in big cats, their body parts and derived products currently occurring in South Korea (57%). Even among respondents who reported that big cat trade still occurred, several noted that this was now much reduced or extremely small in volume.

Several key reasons were identified by expert respondents to explain the reported decline of trade. South Korea’s accession to CITES and subsequent introduction of a trade ban were seen as important, with respondents highlighting perceived strict application of this ban, by customs officials, regarding imported products, and government regulators, regarding the production of traditional medicinal products (n = 7 responses). South Korea’s rapid economic growth in this time period (national GDP per capita tripled between 1994 and 2022) was seen as an important factor in a decline in demand for big cat products (n = 4), because it was linked to the widespread availability of medical services and pharmaceutical medicine in South Korea, as well as a decline in trust in TAMs. Economic growth, when combined with generational turnover and changes in consumer habits, was also considered an important factor in the decline of big cat-derived products as status symbols. In this context, new consumer products, from electronics to luxury cars, were considered far more attractive status symbols. Increased public awareness of the threats to endangered wildlife, linked by respondents to a combination of educational curricula, media attention, and the work of environmental nongovernmental organisations, was also seen as having decreased the social acceptability of trade (n = 7).

Other factors reported to contribute to a decline in trade in big cats, but suggested less frequently, included: advanced urbanisation, which has resulted in declines in South Korea’s rural population and was perceived to have reduced the general accessibility of wildlife products; a decline in demand for big cat skins as fur, as a result of the development of synthetic ‘fake’ fur and increasing awareness of animal welfare issues; and legal regulation of big cat breeding programmes.

The perceived reasons for decline in trade in South Korea given by expert respondents corresponded closely with the recommendations they gave for addressing persistent illegal big cat trade in other countries. Respondents emphasised active participation in CITES and introduction of domestic bans on big cat trade if not already in place; the importance of stringent customs regulation and enforcement of trade bans; and increasing public awareness of bans and the reasons for them, for example through national education systems. Respondents also placed emphasis on general economic development and extending the coverage of medical care, to support demand reduction.

Although perceived strict application of the 1994 trade ban was regularly suggested to be an important factor in the decline of trade (mentioned by 64% of South Korean respondents), few individuals had any information on confiscations or arrests being made in connection with wildlife trade related offences concerning big cats. Several respondents referred to a case from 2010 where a suspected smuggler of tiger skins was arrested by the Seoul Metropolitan Police Agency. One respondent referenced an incident in November 1996, where Seoul Customs officials found two leopard skins and a large consignment of ivory within an air freight shipment from Gabon. Information was also provided regarding the arrests of a Chinese national in 2013 in Weihai, China, who had smuggled leopard skins and ivory from Kenya on a flight that had transferred in Incheon, South Korea [47] and a Vietnamese national in 2020 in Vladivostok, Russia, who had attempted to board a flight to Incheon with pieces of tiger mandible (with canines attached) and a bear gallbladder [48].

### Involvement in trade in other Asian countries

Although the survey did not explicitly discuss trade in other countries involving South Korean nationals, one respondent identified a case involving the arrest and conviction of a South Korean national in China in 2012, for illegally purchasing and reselling bones which were confirmed to be tiger following DNA analysis [49]. Two respondents also mentioned reports of South Korean tourists purchasing tiger-derived products in Thailand and Vietnam (tiger bone wine and tiger bone glue). This was supported by Environmental Investigation Agency investigations in Thailand in 2019, which suggested that groups of South Korean tourists were being taken to retail locations offering tiger-derived products, which they were then encouraged to purchase [50]; the retailers claimed that the source of these products was captive tiger, but the sale of captive tiger parts and products is still prohibited by domestic legislation in Thailand [51]. The scale of this purchase and associated consumption is unknown, as is whether any of these products were carried to South Korea, either for personal use or resale, or were mail ordered. However, one respondent specifically mentioned that they did not believe these purchases were for trade in South Korea and noted that blog posts written by Korean tourists about their experience of trying tiger products in Vietnam seemed to suggest the authors were surprised that such products were available.

### Big cat trade and North Korea

This study deals primarily with trade in big cat body parts and derived products involving South Korea. However, given that until 1945 the Korean Peninsula was a single country, the questionnaire asked respondents whether they had any information on trade in big cats in the Democratic People’s Republic of Korea (henceforth, North Korea). Big cat trade in North Korea is of particular interest because it is one of only a few countries that are not currently Parties to CITES, while the status of its native tiger and leopard populations is uncertain [52].

Of the five respondents who gave information on trade in North Korea, three were able to provide some evidence to support their observations. This included products reported for sale in either China or South Korea and described as originating from North Korea: a leopard skin from China that had been purchased from a North Korean smuggler in the 1990s; tiger bone wine for sale in South Korea in the early 2000s and advertised as having been produced by Pyongyang Central Zoo; and several instances since 2015 of tiger bone wine, reportedly produced in North Korea, for sale in China. In some cases (for example, the leopard skin confiscated in China), respondents noted scepticism regarding the product’s authenticity, either in terms of the species or country of origin.

Further evidence came from seizures in China of products linked to North Korea. This included four confiscations of tiger bone wine between 2011 and 2019, including in Dandong and Tonghua, close to the China-North Korea border, as well as one case of an individual from China’s Korean ethnic minority who was convicted in 2012 in Jilin province, China, for involvement in drug and tiger bone trade [53]. The individual and their accomplices admitted to having crossed the Yalu (Amrok) river into North Korea on several occasions between May and July 2010 in order to obtain 9–10 kg of suspected tiger bone and one tiger skin, and to subsequently return two bones which they believed were not tiger, as well as the tiger skin, presumably due to failure to find a buyer. In September 2011, police raided a property linked to the group and seized 25 suspected tiger bones. On testing, these were found to comprise 0.54 kg of tiger bone, alongside 0.97 kg of lion bone, 2.66 kg of black bear (understood to be *Ursus thibetanus*) bone, and 2.95 kg of brown bear (*Ursus arctos*) bone. Very little is known about wildlife trade between North Korea and China, and further research is needed to understand where traded products came from, given that this case apparently involved one non-native species (lion), as well as several species of currently unknown status and distributional range in North Korea (tiger, black bear, and brown bear).

## Discussion

Understanding potential changes in big cat trade in South Korea over the past 30 years is an important first step towards understanding and combatting any illegal trade that may persist to this day. It also enables lessons to be learnt that may benefit ongoing efforts to address trade in other consumer countries. The overall low number of respondents who felt they had sufficient relevant information to answer the survey, despite having relevant areas of expertise (for example, expertise in trade in Asian big cats, or CITES management in South Korea), indicates significant uncertainty in respect of big cat trade in South Korea over the past 30 years. The covert nature of illegal trade means that absence of information alone does not provide sufficient evidence to conclude absence of trade. Our analysis of expert opinion on big cat trade in South Korea largely coalesced around the position that trade has dramatically reduced since the country’s accession to CITES (1993) and introduction of a big cat trade ban (1994). While there was broad agreement, especially among respondents who worked in South Korea, regarding overall trends, there was no clear consensus on whether trade still occurred in South Korea. However, evidence that some respondents were able to provide was sufficient to show that trade in big cat body parts and derivatives has not entirely collapsed, with examples of small-scale, individual or opportunistic trade being identified. This encompassed domestic trade, as well as trade in other countries, with products then subsequently imported into South Korea. There is sufficient evidence to suggest that some of this trade was illegal.

Despite all Asian big cats being listed by CITES under an Appendix I classification and being subject to CITES Resolution 12.5 (Rev. CoP18) *Conservation of and trade in tigers and other Appendix-I Asian big cat species*, there are some circumstances where international trade in big cats may be permissible. These encompass specific purposes (notably, scientific, breeding, or educational purposes), specific species (African lion is listed under CITES Appendix II) and specific sources (for example, trade in animals which are usually subject to Appendix I listing, but which are captive bred, is permitted under Appendix II). After several years during which there were no CITES records of legal trade in big cat bodies, body parts or derivatives inbound to South Korea (1998–2003), there have been small amounts of CITES-registered international trade most years since 2008 (except 2015, 2017 and 2020), particularly in big cat bodies, but also including trophies, powder, bones, derivatives, extract, skulls and rugs. However, our results illustrate that CITES records may hide some illegal trade, with further inquiry having revealed that a number of records referred to big cat products that were illegally exported from South Korea to the United States and New Zealand (including two shipments that contained a large quantity of tiger-derived products). This list should not necessarily be considered comprehensive, as these two countries are unusual in having historically reported records of illegal trade to CITES.

The source and authenticity of the big cat products identified for sale on different Korean online marketplaces, following leads provided by respondents, is difficult to ascertain with any certainty and an advert may not necessarily have a product behind it. Kang and Phipps [31] reported that a large quantity of tiger bone (1883 kg), over 20% of the total imported by South Korea from 1970 to 1993, was imported in 1993, the year prior to the trade ban. However, while there was a short transition period, use of this pre-1994 tiger bone for pharmaceutical products would now be considered illegal. Although trade in other products derived from pre-convention stock could theoretically be legal, if the manufacturer or seller could prove acquisition prior to 1994, the certification required to do so and comply with the Wildlife Protection and Management Act (2005) is now strictly regulated by the Ministry of Environment. In a number of cases, particularly examples involving individual bottles of tiger bone wine, the sellers described that the product they had listed was either gifted to them in the past or inherited, implying that the item may predate the 1994 trade ban and may have originally been obtained legally. However, domestic trade in these items would still contravene current legislation. In other examples, we have strong reason to believe products were illegally imported into South Korea. For example, the expert report of the skin of a snow leopard (a CITES Appendix I species that is not native to South Korea and is not commonly captive bred) from 2005, which the owner claimed to have obtained in Nepal, does not appear on CITES import records for South Korea and was likely illegally smuggled into the country.

While this survey specifically dealt with big cat trade in South Korea, several respondents suggested the need for greater attention to cases of big cat trade in other countries involving South Korean nationals. One reported case, that of a South Korean national convicted in China for illegally purchasing and reselling tiger bones [49], would seemingly suggest the individual involved had scienter. Such reports are extremely unusual in the literature, but Oswell [51] recorded that during wildlife market surveys conducted in 2008 in Tachilek, on the Myanmar (Burma)-Thailand border, a trader stated that many of his customers were international, especially from Korea and Taiwan, and came specifically to Tachilek to purchase big cat products. Such reports are worth serious consideration as, if accurate, they suggest individuals engaged in travel to specific locations for the purpose of trade. In other examples however, such as reports of Korean tourists being taken to locations where tiger-derived products were available and being encouraged to purchase them, it is far from clear that the tourists involved had prior awareness of the availability of tiger-derived products, or of the legal status of tiger trade.

Although we caution against complacency, given documented evidence that some big cat trade still occurs in South Korea, there are lessons to be learnt from the country’s experience of implementing a trade ban over a short time period. Previous studies have argued that efforts to address demand for endangered species in countries or cultures in which a specific product has been used for a long time may make slow progress. Thomas-Walters et al. [54] highlighted the example of ivory trade in China, noting that it has existed for thousands of years and remains persistent, despite extensive conservation efforts. While we agree that the specific cultural context in which trade operates is important in determining the effectiveness of conservation initiatives focussed on behaviour change, our results show that a long tradition of trade in a particular species (or substitutes for it) does not automatically imply that this cultural use is fixed. Korea has an extremely long history of trade in tiger that stretches back hundreds, and possibly thousands, of years. Despite this, expert opinion suggests that within a 30-year time period, between 1993 and 2023, South Korean trade in tiger declined dramatically. South Korea is also not considered to be an important destination for international lion bone trade, a prominent substitute for tiger [34]. This poses several questions: why was South Korea apparently able to succeed in implementing a big cat trade ban, and what lessons might there be for other countries seeking to address recalcitrant trade?

While our results show that trade bans should not be seen as a panacea, perceived strict implementation (n = 7 responses) and good awareness of the trade ban (n = 7) were two of the most commonly cited reasons for the perceived decline in big cat trade since the 1990s. Expert perceptions of good awareness of the trade ban are supported by Kang and Phipps’ survey of TKM practitioners, in which they found 88.7% of 256 respondents were aware of the ban regarding tiger bone [31]. Given a lack of evidence that the trade ban was any more rigorously enforced than bans which have been introduced in other countries (for example, through seizures or court cases), it is difficult to verify that particular claim with any certainty. However, if it was perceived as such, that may help explain the suspected compliance noted by respondents. Participants suggested that enforcement concerning cross-border trade may benefit from specific obstacles to the supply of big cat products to South Korea, notably that the Korean Demilitarized Zone (DMZ) acts as a ‘hard’ border to the movement of goods. However, while this contrasts with the more porous land borders of several other important consumer countries [55] and means that wildlife contraband must arrive or depart by air or sea, South Korea is not unique in this regard [56,57].

Another potential explanation for reported compliance with the trade ban may be the social stigma towards consumption that bans can induce [58], which may play a particularly important role in South Korea [59]. This explanation provides a potential link with another common observation, that increased public awareness of the threats to endangered wildlife had decreased the social acceptability of trade in big cat products (n = 7). While it is difficult to independently evaluate the validity of this assessment, given an absence of baseline data, Kang and Phipps [31] did find that a large majority of TKM practitioners they surveyed were conscious that the use of banned medicinal materials could negatively impact biodiversity.

As well as explanations directly linked to the trade ban itself, expert respondents identified South Korea’s continued economic growth as an important factor in the decline of big cat trade (n = 4). Economic growth in South Korea has been accompanied by swiftly rising living standards, with widespread availability of contemporary scientific medicine, very high levels of tertiary education, and ready consumer access to alternative luxury goods, which were all identified by expert respondents as reasons for the declining desirability of big cat products. While other studies have linked economic growth in historic consumer countries in East and Southeast Asia with growth in demand for tiger products [60], our results suggest that this outcome should not be a universal assumption. However, we caution against using economic growth as a sole explanation for perceived decline in big cat trade in South Korea, given that the country also experienced strong economic growth between the 1960s and early 1990s [61,62], when trade in tiger was high [29,30].

Parallels can be drawn between the reported rapid decline in big cat trade in South Korea and the collapse of trade in big cat skins in European countries and the United States after CITES Appendix I listing and the introduction of trade bans, which were subsequently extended to smaller felids, in the 1970s and 1980s [1,32,63]. This collapse in trade in Europe and the United States also coincided with well-publicised and rigorously implemented trade bans, high levels of economic development, and changes in consumer preferences linked to the impact of pressure group campaigns [32].

Finally, we reflect on the overlooked nature of wildlife trade in South Korea, as illustrated by the uncertainty surrounding big cat trade over the past 30 years. This issue is not restricted to felids. Previous studies have shown that wildlife trade in South Korea in other taxa groups, including the Ursidae [64] and Mustelidae [59], has historically been overlooked by researchers and policy makers. As highlighted by the findings and limitations of this study, an important consequence is that the pool of experts on illegal wildlife trade in South Korea is relatively small. Increasing capacity in this area could help South Korea to identify and respond to current and emerging trade-based threats, and would benefit future assessments that draw on expert opinion.

## Conclusions and recommendations

We assess changes in big cat trade in South Korea since the country joined CITES (1993) and introduced a ban on trade in endangered big cats (1994), drawing on an expert elicitation survey, expert-led evidence and CITES trade records. Expert opinion suggests that big cat trade linked to South Korea has likely substantially reduced since 1994, but that trade has not stopped altogether. Alongside an increased volume of CITES-registered legal trade since 2008, some opportunistic, individual trade continues to occur, both within South Korea and involving big cat contraband being illegally imported into, or exported from, the country. In order to better understand and address continuing levels of illegal trade we make the following recommendations.

*For policymakers*, *officials and conservationists in South Korea*:

- While South Korea appears to have made good progress on big cat trade, policymakers and law enforcement should exercise vigilance regarding the potential ongoing occurrence of illegal trade. Efforts to ensure that illegal international trade is identified and intercepted should be conscious of the potential entry of products into South Korea, the transit of products enroute to other markets, and products being exported from or taken out of South Korea.
- The South Korean government should investigate any online product pages or posts promoting the sale of tiger- and other Asian big cat-derived products, working with the relevant platforms to remove them. Despite previous advice to do so by the Environmental Investigation Agency [50], big cat products are still being publicly sold online.
- South Korean government agencies, potentially supported by relevant non-governmental organisations, should implement regular monitoring and surveillance of open, and any potential hidden, big cat trade. Consumer attitude and demand surveys may reveal information on the motivations and perspectives of individuals involved in ongoing trade, which could help inform targeted demand-reduction campaigns.
- To support the implementation of these recommendations, South Korea would benefit from the establishment of a dedicated unit within a public body, or wider group integrating research institutes and non-governmental organisations, to: (i) identify and monitor illegal wildlife trade and other forms of wildlife crime, and (ii) provide intelligence, advice and technical support to government and law enforcement agencies. This unit could also provide coordinating functions at a national level and serve as a focal point for international collaboration.
- South Korean tour companies and travel agencies should be made aware that purchasing tiger products in Thailand and Vietnam is illegal, and should feed this information back to their customers. Targeted engagement with points of contact for travellers (for example, online platforms or social media) could also help raise awareness. Signage regarding illegal wildlife trade, its legal status and its impacts on endangered wildlife should be prominently displayed at Incheon International Airport, South Korea’s primary international airport. Airport staff should receive regular training regarding illegal wildlife trade, including trade in felids, and familiarisation with the tools and resources available through CITES for frontline enforcement personnel.
- South Korean customs and law enforcement would benefit from, and be well placed to contribute to, enhanced regional information-sharing and intelligence analysis, in order to increase detection and interception of international, illegal trade. A range of recommendations for further engagement with existing mechanisms and platforms, as well as the creation of new partnerships, are set out in the CITES Big Cats Task Force 2023 Meeting Notes [65]. Any instances of international trafficking should be reported to the INTERPOL National Central Bureau (NCB) in Seoul.

For international conservation organisations and wildlife trade specialists:

- Increased engagement between international conservation organisations and wildlife conservation in South Korea would help continue to build national capacity to combat illegal trade. This may include providing independent support and constructive challenge to government; supporting the development of local expertise; and sharing lessons between South Korea and other countries. This could be achieved through the establishment of local offices or programmes, or partnerships with South Korean organisations.
- Finally, reports of North Korean products reputed to be derived from big cats are worth further investigation, to determine the authenticity of these products, their origin, and details of their trade. While a long-term view to CITES accession for North Korea would be beneficial, the CITES secretariat should be mindful of the broad sanctions currently in place on North Korea.
